# Body Habitus Impact on Success of Cryoneurolysis for Postoperative Total Knee Arthroplasty Pain Control: A Retrospective Cohort Study

**DOI:** 10.1016/j.artd.2023.101164

**Published:** 2023-07-24

**Authors:** Brenton R. Jennewine, Clayton W. Wing, William M. Mihalko

**Affiliations:** Department of Orthopaedic Surgery and Biomedical Engineering, University of Tennessee Health Science Center-Campbell Clinic, Memphis, TN, USA

**Keywords:** Cryoneurolysis, Body habitus, Total knee arthroplasty

## Abstract

**Background:**

Cryoneurolysis utilizes temperatures below −20°C for nonpermanent analgesia to control pain in total knee arthroplasty (TKA). There is concern that body habitus could limit pain control because of accuracy of cryoneurolysis to subcutaneous nerves. This study aimed to determine the relationship between body habitus and effectiveness of cryoneurolysis on postoperative pain control.

**Methods:**

A retrospective chart review was performed on patients undergoing cryoneurolysis before primary TKA from 2017 to 2019. Included were 114 patients (58 control group and 56 treatment group). Cryoneurolysis patients were divided into 3 groups (small, medium, and large) based on the soft tissue to femoral diaphysis ratio of 7 cm proximal to superior pole of the patella. Postoperative outcome measures were morphine equivalents, numerical rating score for pain, range of motion, and Knee Injury and Osteoarthritis Outcome Score Joint Replacement.

**Results:**

The small cryoneurolysis group showed decreased opioid consumption at the 2, 6, and 12 weeks compared with control group, with morphine equivalents significantly decreased at 2 weeks for small compared with medium groups (54.3 vs 142.9, *P* = .0097). Numerical rating score for pain decreased significantly between small and medium groups (3.4 vs 4.0, *P* = .012) and between medium and large groups (4.0 vs 2.4, *P* = .012). Range of motion increased at 12 weeks for small group compared with medium group (118 vs 112, *P* = .042). There were no differences in any outcome measure between small and large groups.

**Conclusions:**

Body habitus does not appear to affect efficacy of cryoneurolysis in controlling postoperative pain following TKA. Cryoneurolysis remains a useful tool for multimodal pain management.

## Introduction

As the population ages, demand for total knee arthroplasty (TKA) is increasing. With nearly 700,000 primary TKAs performed in the United States in 2012, this number is expected to grow by more than 140% by the year 2050 [1]. With the increase in demand, strategies to minimize postoperative pain and maximize the speed of recovery will be necessary to meet this increasing need. The use of opioid pain medication originally was a primary component of controlling postoperative pain, but with the persistent opioid crisis, the need to minimize the use of opioid medications is imperative [2,3]. With the relatively recent adoption of multimodal pain management strategies that target numerous pain pathways, the use of opioids for pain control following total joint replacements has diminished [4]. Despite these advances, narcotic pain medication usage remains an issue [5].

More recently, orthopaedic surgeons have begun to use additional strategies to manage pain in a multimodal fashion, including periarticular injections and peripheral nerve blocks [6]. Cryoneurolysis is a recent addition to the field of peripheral nerve blocks that utilizes temperatures below −20°C to cause nonpermanent analgesia via Wallerian degeneration of the nerve axons [[7], [8], [9]]. Multiple studies have demonstrated the effectiveness of cryoneurolysis in both decreasing pain and the use of opioids following surgery [[10], [11], [12]]. In the application of this technique to TKA, the targets for cryoneurolysis are the superficial genicular nerves, including the infrapatellar branches of the saphenous nerve (ISN) and anterior femoral cutaneous nerve (AFCN), as these nerves are the main contributors for pain related to the surgical incision and soft tissue damage during TKAs [13].

Since cryoneurolysis is typically performed based on anatomic landmarks, there is concern that body habitus may detract from the accuracy of cryoneurolysis to these nerves, limiting the effectiveness of this treatment for pain. As such, this study seeks to determine the relation between body habitus and postoperative pain following cryoneurolysis and TKA. We hypothesize that an increase in thigh circumference as determined by the soft tissue envelope anterior to the distal femur will correlate with an increase in postoperative pain as well as an increased use of total morphine equivalents (ME).

## Material and methods

### Study design

A retrospective chart review was performed on patients who had previously undergone an unblinded, randomized controlled trial from 2017 to 2019 to evaluate the efficacy and safety of cryoneurolysis treatment before TKA for reducing postoperative opioid use at a single study center [10]. Approval for this study was granted by the institutional review board. Inclusion criteria included patients between the ages of 22 and 79 who were scheduled to undergo primary unilateral TKA due to osteoarthritis and had an anticipated discharge to home following the procedure. Exclusion criteria of the original study were daily or almost daily use of opioids (defined as habitual use of opioids on the basis of clinical judgment) for more than 3 months before enrollment, a concurrent painful physical condition that required analgesic treatment during study follow-up, preoperative varus or valgus deformities greater than 15 degrees, previous cryoneurolysis treatment, body mass index greater than 40 kg/m^2^, previous surgery in the region to be treated, history of a clotting disorder, and anticoagulant medication within 7 days before treatment.

### Study groups

In the original study, patients were randomized into a control group (n = 58) and a treatment group (n = 57) who received preoperative cryoneurolysis. As reported in the prior study, the Iovera device (Pacira CryoTech, Inc., Fremont, CA, USA) was utilized for cryoneurolysis [10]. Cryoneurolysis was performed for all patients in our clinic 3-7 days prior to schedule TKA. Nitrous oxide flows from the Iovera device to the tip of a closed-ended needle, forming a localized cold zone at this tip. The target nerves for this study were the ISN and AFCN, which control much of the anterior pain related to the surgical exposure and soft tissue damage in TKAs. Placement of the Iovera device was based on anatomic reference points, as opposed to ultrasound, in accordance with manufacturer and prior literature specifications [[7], [8], [9]]. The AFCN was targeted by administering a treatment roughly the width of the patella at a point 7 cm proximal to the superior pole of the patella. The ISN was targeted by focusing treatment along a line that began 5 cm medial to the inferior pole of the patella, to a point that was 5 cm medial to the tibial tubercle. Along these 2 separate treatment lines, multiple needle insertions were utilized to fully apply cryoneurolysis to this region. The depth for cryoneurolysis treatment is predetermined by the probe. Per manufacturer specifications, the probe is completely pushed into the skin so that the base of the probe tip is against the skin, as the probe base has a warming plate to prevent skin necrosis.

Beyond this preoperative cryoneurolysis, all patients (ie, control and treatment groups) had similar pain management regimens. Periarticular local analgesia infiltration was only provided in the posterior capsule, as this area of the knee would not receive adequate analgesia from cryoneurolysis of the AFCN and ISN. Postoperative multimodal therapy consisted of 1000 mg acetaminophen (40 pills) 3 times daily, 100 mg gabapentin (40 pills) 3 times daily, 15 mg meloxicam (15 pills) once daily, 50 mg tramadol (40 pills) every 6 hours, and 5 mg oxycodone (40 pills) to be taken as needed for rescue pain relief every 4 hours.

This current study is a subanalysis of the treatment arm of the original patient population investigating how body habitus may affect the accuracy of the cryoneurolysis treatment, given that this cryoneurolysis is performed based on anatomic reference points as opposed to ultrasound visualization of the sensory nerves. In this study, patients within the treatment group were further subdivided based on body habitus based on anterior thigh depth relative to femur diameter. Preoperative lateral knee radiographs were reviewed to assess the anterior soft tissue envelope through which the Iovera device would be utilized to target the AFCN. At a point 7 cm proximal to the superior pole of the patella, the depth of the soft tissue from skin to the femur was recorded, as well as the diameter of the femur at this point. This soft tissue envelope was then normalized to femur diaphysis by dividing the depth of the soft tissue by the femoral diameter, creating a standardized ratio for comparison between patients of a small (ratio anterior soft tissue to femoral diaphysis <1.3), medium (ratio between 1.4 and 1.7), and large (ratio >1.7) thigh measurements.

### Patient follow-up and assessments

Study patients were seen postoperatively at 72 hours, 2 weeks, 6 weeks, and 12 weeks. At these visits, the following patient outcomes were reported: ME from oxycodone usage, numerical rating scale (NRS) of patient pain in the past week and currently (NRS-now), range of motion (ROM), Knee Injury and Osteoarthritis Outcome Score Joint Replacement (KOOS JR), and use of an assistive device. Preoperatively, similar patient outcomes were reported in addition to the standard patient demographic data.

### Statistical analysis

As discussed in our prior research, sample size within the original patient population was determined on the basis of providing at least 80% power for the primary endpoint using a 1-sided, 2-independent sample Satterthwaite *t*-test to test for superiority of cryoneurolysis vs standard of care treatment with a significance level of 0.025, assuming a 1:1 allocation to treatment, a true treatment effect of −12.0 mg, and true standard deviations of 16.9 and 26.0 mg for the cryoneurolysis and standard of care treatments, respectively. A sample size of 57 patients was determined to adequately power the study.

Analysis of the subdivided treatment groups (small, medium, and large) compared with the control group was performed with a 1-sided *t-*test with a significance level of 0.05. Further analysis of the individual subdivided treatment groups was performed with one-way analysis of variance to compare the outcomes between the small, medium, and large groups, and the *P*-value for significance was set at 0.05. If there was a significant *P*-value, then a Tukey-Kramer post hoc test was performed to identify which values between specific groups held significance. The Tukey-Kramer test was used post hoc instead of many student *t*-tests because it accounts for variances in all groups at the same time and helps control the family-wise error rate.

## Results

### Patient demographics

A total of 56 patients were analyzed in this study between the 3 different treatment groups. There were 17 patients in the small group, 21 in the medium group, and 18 in the large group. Additionally, a total of 58 patients were included in the control group. Patient characteristics were overall similar between treatment groups, excluding a difference in gender distribution between the groups, increased prevalence of diabetes in the large group, and increased prevalence of alcohol use in the small group. These characteristics can be reviewed further in Table 1.

**Table 1:** Patient characteristics of the cryoneurolysis treatment groups.

Demographic	Control	Cryoneurolysis body habitus subgroup		
		Small	Medium	Large
Age, y (SD)	65.2 (9.0)	68.06	65.76	64.78
BMI (SD)	31.8 (5.4)	28.1	30.64	31.0
Gender, n (%)				
Male	26 (44.8)	14 (82.4)	12 (57.1)	5 (27.8)
Female	32 (55.2)	3 (17.6)	9 (42.9)	13 (72.2)
Diabetes (%)	10.3	5.8	14.2	22.2
Stroke (%)	0	0	5	0
Mood disorder (%)	13.8	17.6	9.5	16.7
Tobacco use (%)	8.6	35.3	23.8	33.3
Alcohol use (%)	37.9	52.9	42.9	22.2

SD, standard deviation.

### Opioid consumption

The primary endpoint of this study investigated whether opioid use at 3 days, 2, 6, and 12 weeks after primary TKA as documented by ME taken since the prior follow-up appointment was dependent upon the size of the patient’s thigh. At the 2-week visit, the small group had a decrease in opioid usage compared with the medium group (ME 54.3 vs 142.9, *P* = .0097), but there was no significant difference between the small and large groups at this time point (Table 2). Total ME were not significantly different between the 3 body habitus groupings at the 72-hour, 6-week, and 12-week postoperative visits, indicating that there was no linear correlation between opioid usage and the body habitus of the patient's thigh.

**Table 2:** Comparison of opioid consumption in total morphine equivalents of control and cryoneurolysis treatment groups; mean (SD).

Follow-up visit	Control	Cryoneurolysis body habitus subgroup		
		Small	Medium	Large
72-h	56.4 (48.5)	37.5 (45.3)	42.1 (34.8)	35 (28.9)
2-wk	91.8 (81.2)	54.3 (69.6)^a^^b^	142.9 (101.8)^a^^b^	83.8 (92.7)
6-wk	58.4 (77.7)	16.8 (35.6)^b^	64.5 (172.1)	42.1 (83.8)
12-wk	30.3 (54.6)	1.3 (3.2)^b^	2.0 (6.2)^b^	28.9 (86.3)

a Significant difference between cryoneurolysis treatment group as identified by analysis of variance (ANOVA) and Tukey-Kramer post hoc testing.

b Significant difference between control and cryoneurolysis treatment group as identified by student t-test.

When comparing the cryoneurolysis treatment subgroups to the control group, ME were lower for the small group (2-week, 6-week, and 12-week visits), medium group (12-week visit), and large group (72-hour visit), which were statistically significant. The remainder of this data can be found in Table 2.

### Secondary outcomes

Several secondary endpoints were also evaluated to determine the effectiveness of cryoneurolysis with varying body habitus. These included NRS-now and NRS of patient pain in the past week, ROM, and functional outcomes as measured by KOOS JR. Significance was found in ROM between the small and medium groups at the 12-week visits (118 degrees vs 112 degrees, *P* = .042). Additionally, NRS-now scores were significantly different between the small and medium groups at the 2-week visit (3.4 vs 4.0, *P* = .012), as well as between the medium and large groups (4.0 vs 2.4, *P* = .012).

Comparing the cryoneurolysis treatment subgroups to the control group, significantly increased ROM was seen in the small group at the 72-hour and 12-week follow-ups and within the large group at the 2- and 6-week follow-up visits. With regard to pain NRS scores, significant decreases were seen at various follow-up visits in all 3 subgroups, as shown in Table 3. KOOS JR scores were significantly increased at the 72-hour follow-up visit for medium and large groups, and at the 2-week visit for all 3 subgroups. The remainder of the data comparing the control group and treatment groups with regard to secondary outcomes can be found in Table 3.

**Table 3:** Comparison of secondary outcomes of control and cryoneurolysis treatment arms; mean (SD).

Follow-up visit	Control group	Cryoneurolysis body habitus subgroup		
		Small	Medium	Large
72-h				
ROM	85.3 (15.7)	96.6 (15.3)^a^^b^	82.6 (19.2)^a^	86.1 (15.7)
NRS-now	5.4 (2.1)	4.3 (1.9)	4.2 (2.3)^b^	4.3 (2.5)^b^
NRS-7	6.0 (2.2)	5.9 (2.0)	5.7 (2.0)	5.1 (1.7)
KOOS JR	57.0 (9.6)	53.6 (13.8)	63.3 (6.6)^b^	62.5 (7.2)^b^
2-wk				
ROM	96.5 (12.0)	100.6 (11.6)	86.3 (15.3)	102.4 (10.4)^b^
NRS-now	3.3 (2.6)	3.4 (2.0)^a^	4.0 (2.4)^a^	2.4 (1.5)^a^
NRS-7	4.4 (2.3)	4.9 (2.1)	4.4 (1.5)	36 (1.7)
KOOS JR	63.2 (9.0)	65.5 (8.0)^b^	67.3 (7.8)^b^	69.5 (8.9)^b^
6-wk				
ROM	108.1 (10.1)	110.6 (12.1)	108.5 (11.2)	113.2 (9.4)^b^
NRS-now	1.9 (2.1)	1.7 (2.0)	2.1 (2.0)	1.6 (1.6)
NRS-7	2.8 (2.2)	2.4 (2.1)	2.4 (2.0)	1.7 (1.5)^b^
KOOS JR	69.2 (10.5)	71.1 (10.1)	72.3 (8.3)	71.2 (10.1)
12-wk				
ROM	111.9 (9.1)	117.9 (6.9)^a^^b^	111.6 (5.1)^a^	114.0 (9.3)
NRS-now	1.5 (1.9)	0.8 (1.0)	1.4 (2.5)	1.0 (1.7)
NRS-7	2.3 (2.2)	1.4 (1.3)^b^	1.6 (2.4)	1.2 (1.7)^b^
KOOS JR	74.4 (11.9)	76.5 (10.5)	78.7 (14.4)	77.9 (9.4)

KOOS JR, Knee Injury and Osteoarthritis Outcome Score Joint Replacement; NRS-now, pain numerical rating score now; NRS-7, pain numerical rating score in last 7 days; ROM, range of motion.

a Significant difference between cryoneurolysis treatment group as identified by analysis of variance (ANOVA) and Tukey-Kramer post hoc testing.

b Significant difference between control and cryoneurolysis treatment group as identified by student *t*-test.

## Discussion

Perioperative multimodal pain control strategies are continually being improved upon in order to decrease patient consumption of opioids and optimize patient pain, especially as outpatient total arthroplasty is utilized more consistently. To this end, preoperative cryoneurolysis has been shown to be a novel approach to multimodal pain control in TKA [9,10]. Prior studies have indicated that this technique results in the reduction of opioid use following TKA, possibly limiting narcotic dependence [10,[12], [13], [14], [15], [16]]. Similar to this present study and prior literature, cryoneurolysis is performed via an anatomic landmark approach with effective treatment of the ISN and AFCN [10,[12], [13], [14], [15], [16]]. Given the anatomically based technique for administering the Iovera cryoneurolysis to the ISN and AFCN, one would suspect that increased body habitus would negatively affect the anesthetic results due to the depth of the sensory nerves from the surface of the skin. Interestingly, the effectiveness of the cryoneurolysis utilizing opioid consumption as a surrogate for pain control did not linearly correlate with changes in body habitus.

Regarding opioid consumption between the control and treatment subgroups, there were some statistically significant decreases in consumption in all 3 body habitus groups; however, the small group showed a consistent trend in decreased consumption at the 2-, 6-, and 12-week follow-up visits. As stated previously, the prior study that utilized this data set showed a statistically significant decrease in opioid consumption with preoperative cryoneurolysis treatment; however, the treatment arms were not divided into body habitus subgroups in this initial analysis. These smaller treatment groups may be underpowered to show significance as the treatment groups themselves trended toward lower opioid consumption compared with the control group.

Current literature does support the addition of cryoneurolysis into a multimodal pain management plan for TKA, similar to our previously reported results [10]. Lung et al. [14] found that preoperative cryoneurolysis resulted in decreased opioid morphine milligram equivalents consumed by patients compared with a control group. While the opioid consumption results were not statistically significant, likely due to small and underpowered sample sizes, they also found that patients treated with cryoneurolysis had significantly improved postoperative ROM, ambulation distance, and KOOS JR scores, which may reflect an overall improvement in patient satisfaction and pain [14]. Urban et al. [15] similarly investigated preoperative cryoneurolysis on 169 patients prior to TKA, finding that the cryoneurolysis group had significantly decreased opioid consumption by 51%, had lower mean pain scores by 22%, and had a reduced length of inpatient stay.

When comparing the 3 body habitus treatment groups, there was one statistically significant decrease in morphine equivalent usage between the medium and small group. This occurred at only one time point, and there were no further significant differences between the 3 body habitus groups at any other time point. This indicates there is likely no linear correlation between the patient's body habitus and effectiveness of cryoneurolysis to limit opioid consumption. A similar finding also exists for the secondary outcomes. While there were various statistically significant differences between the 3 treatment groups with regard to the secondary outcomes, these are likely not clinically relevant.

A possible reason for these findings may be related to the design of the Iovera Smart Tip cryoneurolysis applicators and local anatomic considerations. In this study, the AFCN and ISN were localized by using the landmark of one third of the distance from the superior pole of the patella to the anterior superior iliac spine and 5 cm medial to the tibial tubercle, respectively. Thigh depth where the AFCN is targeted ranged anywhere from 26 mm to 99 mm in our patient population, with an average anterior thigh depth of 52 mm. The various Smart Tip options on the Iovera can create surrounding treatment areas measuring anywhere from 5.4 mm by 9.4 mm (width by height) to 7.1 mm by 16.0 mm [7]. This relatively large treatment area, especially in the anterior-posterior/height dimension, may allow for some decreased precision in targeting the sensory nerves caused by variations in body habitus.

Several limitations exist in this current study. First, there were a low number of patients within each body habitus treatment group, likely showing these subgroups were underpowered for full analysis. While the original study population was adequately powered with 57 patients in the control and treatment groups [10], division of the cryoneurolysis treatment group into 3 separate subgroups resulted in low patient numbers within these groups. Larger populations are needed to fully assess the effect body habitus may have on cryoneurolysis treatment efficacy; however, our results did not show any linear correlation at all, which may reflect that an anatomic approach for cryoneurolysis treatment is not influenced by body habitus. Additionally, cryoneurolysis treatment was administered based on anatomic landmarks without visualization of the targeted sensory nerves, which may affect isolation and treatment of these nerves. However, this anatomic landmark technique has been utilized across multiple studies showing effective treatment of the sensory nerves [10,[12], [13], [14], [15], [16]].

## Conclusions

Cryoneurolysis is an innovative approach that may help improve multimodal pain control and minimize opioid use following TKA. This study investigated whether patient body habitus impacted the efficacy of cryoneurolysis in targeting sensory nerves related to TKA postoperative pain. While there was a decrease in opioid consumption at the 2-week postoperative visit between small and medium-sized groups in this study, there was not a linear correlation between body habitus and opioid consumption. Based on these results, body habitus does not appear to have a negative impact on the effectiveness of cryoneurolysis for pain control following TKA.

## Conflicts of interest

W.M. Mihalko reports receiving consulting fees from Pacira Pharmaceuticals, Inc., the manufacturer of the product used in this study, in addition to unrelated fees from Aesculap/B. Braun, the Department of Defense, Medtronic, Myoscience, Inc., National Institutes of Health (NIAMS & NICHD), and Saunders/Mosby-Elsevier. The data used in this study originated from a study in which Pacira BioSciences participated.

For full disclosure statements refer to https://doi.org/10.1016/j.artd.2023.101164.

## Supplementary data

Conflict of Interest Statement for Jennewine

Conflict of Interest Statement for Wing

Conflict of Interest Statement for Mihalko

## References

[1] Inacio M.C.S., Paxton E.W., Graves S.E., Namba R.S., Nemes S. (2017). Projected increase in total knee arthroplasty in the United States – an alternative projection model. Osteoarthritis Cartilage.

[2] Li J.-W., Ma Y.-S., Xiao L.-K. (2019). Postoperative pain management in total knee arthroplasty. Orthop Surg.

[3] Lespasio M.J., Guarino A.J., Sodhi N., Mont M.A. (2019). Pain management associated with total joint arthroplasty: a primer. Perm J.

[4] Memtsoudis S.G., Poeran J., Zubizarreta N., Cozowicz C., Morwalk E.E., Mariano E.R. (2018). Association of multimodal pain management strategies with perioperative outcomes and resource utilization: a population-based study. Anesthesiology.

[5] Cancienne J.M., Patel K.J., Browne J.A., Werner B.C. (2018). Narcotic use and total knee arthroplasty. J Arthroplasty.

[6] Elmallah R.K., Chughtai M., Khlopas A., Newman J.M., Stearns K.L., Roche M. (2018). Pain control in total knee arthroplasty. J Knee Surg.

[7] Iovera u. https://www.iovera.com/hcp/12265_rev_e_092018.pdf.

[8] Zhou L., Kambin P., Casey K.F., Bonner F.J., O’ Brien E., Shao Z. (1995). Mechanism research of cryoanalgesia. Neurol Res.

[9] Gabriel R.A., Ilfield B.M. (2018). Novel methodologies in regional anesthesia for knee arthroplasty. Anesthsiol Clin.

[10] Mihalko W.M., Kerkhof A.L., Ford M.C., Crockarell J.R., Harkess J.W., Guyton J.L. (2021). Cryoneurolysis before total knee arthroplasty in patients with severe osteoarthritis for reduction of postoperative pain and opioid use in a single-center randomized controlled trial. J Arthroplasty.

[11] Ilfield B.M., Gabriel R.A., Trescot A.M. (2017). Ultrasound-guided percutaneous cryoneurolysis providing postoperative analgesia lasting many weeks following a single administration: a replacement for continuous peripheral nerve blocks?: a case report. Korean J Anesthesiol.

[12] Radnovich R., Scott D., Patel A.T., Olson R., Dasa V., Segal N. (2017). Cryoneurolysis to treat the pain and symptoms of knee osteoarthritis: a multicenter, randomized, double-blind, sham-controlled trial. Osteoarthritis Cartilage.

[13] Horner G., Dellon A.L. (1994). Innervation of the human knee joint and implications for surgery. Clin Orthop Relat Res.

[14] Lung B.E., Karasavvidis T., Sharma A.K., Amirhekmat A., Stepanyan H., McMaster W. (2022). Cryoneurolysis is a safe, effective modality to improve rehabilitation after total knee arthroplasty. Life.

[15] Urban J.A., Dolesh K., Martin E. (2021). A multimodal pain management protocol including preoperative cryoneurolysis for total knee arthroplasty to reduce pain, opioid consumption, and length of stay. Arthroplast Today.

[16] Dasa V., Lensing G., Parsons M., Harris J., Volaufova J., Bliss R. (2016). Percutaneous freezing of sensory nerves prior to total knee arthroplasty. Knee.

