# Supplementary material for: Body Habitus Impact on Success of Cryoneurolysis for Postoperative Total Knee Arthroplasty Pain Control: A Retrospective Cohort Study
Source: Arthroplast Today. 2023 Jul 24;22:101164. doi: 10.1016/j.artd.2023.101164 (PMC10382622; doi:10.1016/j.artd.2023.101164)
Supplement: Conflict of Interest Statement for Mihalko [file mmc3.pdf]

CONFLICT OF INTEREST STATEMENT

American Association of Hip and Knee Surgeons

(Adopted from the American Academy of Orthopaedic Surgeons disclosure statement)

The following form **must be filled out completely and submitted by each author (example, 6 authors, 6 forms). All items require a response. If there is no relevant disclosure for a given item, enter "*None*."**

Body habitus impact on success of cryoneurolysis for postoperative total knee arthroplasty pain control: a retrospective cohort study

Manuscript Title

Royalties from a company or supplier (The following conflicts were disclosed)

Aesculap/B.Braun

2. Speakers bureau/paid presentations for a company or supplier (The following conflicts were disclosed)

Aesculap/B.Braun, Pacira Biosciences, Inc.

3A. Paid employee for a company or supplier (The following conflicts were disclosed)

3B. Paid consultant for a company or supplier (The following conflicts were disclosed)

Pacira, Inc.

3C. Unpaid consultants for a company or supplier (The following conflicts were disclosed)

4. Stock or stock options in a company or supplier (The following conflicts were disclosed)

Medtronic

5. Research support from a company or supplier as a Principal Investigator (The following conflicts were disclosed)

Aesculap/B. Braun; Department of Defense; Myoscience, Inc.; National Institutes of Health (NIAMS & NICHD)

6. Other financial or material support from a company or supplier (The following conflicts were disclosed)

7. Royalties, financial or material support from publishers (The following conflicts were disclosed)

Saunders/Mosby-Elsevier

8. Medical/Orthopaedic publications editorial/governing board (The following conflicts were disclosed)

Journal of Arthroplasty; Journal of Orthopaedic Research; Orthopedic Clinics of North America; The Journal of Long-Term Effects of Medical Implants

9. Board member/committee appointments for a society (The following conflicts were disclosed)

American Association of Orthopaedic Surgeons; American Association of Hip and Knee Surgeons; American Society For Testing Materials International; The Campbell Clinic Foundation; Hip Society; Knee Society; Orthopaedic Research Society

Each author must sign AND print or type his/her name, date and submit a separate form

In addition, one BLINDED Conflict of Interest form (no author names used) should be submitted per manuscript with all author disclosures.

William M. Mihalko, MD, PhD, FAAOS	*William M. Mihalko, MD, PhD*	8/29/22
Author Name (Print or Type)	Author Signature	Date
